# Supplementary material for: Evaluation of Diet and Symptom Severity in Disorder of Gut–Brain Interaction
Source: J Clin Med. 2024 Jul 15;13(14):4132. doi: 10.3390/jcm13144132 (PMC11277627; doi:10.3390/jcm13144132)
Supplement: Supplementary file 1 [file jcm-13-04132-s001.zip › jcm-3057931-supplementary.pdf]

Evaluation of the diet of people with gastrointestinal disorders

Dear Participants,

Before you begin filling out the survey, we would like to assure you that your participation in the study is completely voluntary and anonymous. Please read the following information and begin filling out the survey, which will be tantamount to agreeing to participate in our study. Your cooperation is extremely valuable to our research, however, we retain full assurance of anonymity. You may stop completing the survey at any time, with no consequences for you. Thank you for your involvement.

METRICS

Gender:

□ Female

□ Male

Age:..............................

Height..............................(cm)

Body weight:........................(kg)

Education:

□ Elementary

□ Secondary

□ Higher

Place of residence:

□ Rural

□ City with less than 50,000 inhabitants

□ City of 50-100,000 inhabitants.

□ City of more than 100,000 inhabitants

1. **Are you experiencing any of the following ?**

Symptoms	Occur frequently	Occur occasionally	Do not occur
Abdominal pain			
Nausea			
Constipation			
Flatulence			
Bouncing			
Diarrhea			
Excessive passing gas			
Overflowing			
Fasting abdominal pain			
Nighttime abdominal pain			
Chronic fatigue and weakness			

2. **Due to the presence of adverse gastrointestinal symptoms there been a change in diet?**
   □ Yes
   □ No
   □ Don't know

3. **Do any of the products specifically aggravate the ailment ?**

Kind of product	Yes	No	Sometimes
Milk and milk products			
Raw vegetables and			

fruits			
Brassica vegetables			
Legumes			
Coffee			
Alcohol			
Products high in fiber content			
Products high in fatty products			
Fried products			
Spicy foods/products			

4. Which products have been eliminated from the menu to reduce discomfort?

□ Milk and dairy products.

□ Coffee

□ Alcohol

□ Carbonated water

□ Citrus fruits

□ Spicy products/foods

□ Fried products

□ Raw vegetables and fruits

□ Legumes

□ No products have been eliminated

5. How many meals do you eat in a day?

□ Less than 3 meals

□ 3 meals

□ 4-5 meals

□ More than 5 meals

□ Don't know

6. How long are the breaks between meals?

□ One hour

□ 1-2 hours

□ 2-3 hours

□ More than 3 hours

□ Don't know

7. How often do you eat fruits and vegetables ?

Frequency	Several times daily	Once a day	3 or more times a week	1-2 times a week	I do not consume
Fruit					
Vegetables					

8. In what form do you most often consume vegetables and fruits ?

	Cooked	Raw	Processed	In the form of juice	I do not consume
Fruit					
Vegetables					

9. How often do you eat meat?

□ Several times a day

□ One time a day

□ 3 or more times a week

□ 1-2 times a week

□ I do not consume

10. What type of meat do you eat most often?

□ White meat (e.g. chicken, turkey, veal)

□ Red meat (e.g. beef, mutton, pork)

□ Offal (e.g. liver, heart)

□ I do not eat meat

11. How often do you eat fish?

□ 1-2 times a week

□ More than 2 times a week

□ Several times a month

□ I do not consume

12. What form of heat treatment do you use most often?

□ Frying

□ Braising

□ Cooking

□ Baking

13. What type of grain products do you consume most often?

Product group	Several Times daily	One time per day	3 or more Times a week	Several Times per month	I do not consume
Whole wheat bread					
Wheat bread					
Coarse-grain groats					
Fine-grained groats					
Wheat pasta					
Whole wheat pasta					
Oat flakes					

14. How often do you eat legumes (chickpeas, peas, beans)?

□ 3 or more times a week

□ 1-2 times a week

□ Several times a month

□ I do not consume

15. How often do you consume dairy products?

Product	Several Times daily	One time per day	3 or more Times a week	1-2 times a month	I do not consume
Non-fermented beverages Dairy products (e.g. cow's milk)					
Fermented dairy drinks (e.g., kefir, yogurt)					
Cottage cheese					
Renneted cheeses (e.g. Brie, camembert, gouda Feta)					

16. How often do you consume coffee?

□ Several times a day

□ Once a day

□ Several times a week

□ Occasionally

□ I do not consume

17. How often do you consume tea ?

□ Several times a day

□ Once a day

□ Several times a week

□ Occasionally

□ I do not consume

18. How many fluids do you consume during the day ?

□ Less than 1l

□ About 1.5l

□ About 2l

□ More than 2l

19. What kind of drinks do you consume most often ?

□ Water (e.g., mineral, spring)

□ Carbonated beverages (e.g. Coca-cola, Sprite)

□ Juices

□ Freshly squeezed juices

20. How often do you consume highly processed foods such as FastFood ?

□ several times a day

□ several times a week

□ occasionally

□ I do not consume

21. How often do you consume sweets ?

□ several times a day

□ 3 or more times a week

□ Occasionally

□ I do not consume

22. Do you consume alcohol?

□ Yes, every day

□ Yes, several times a week

□ Occasionally

□ I do not consume

23. What type of fat do you use most often ?

□ Butter

□ Margarine

□ Vegetable oils (e.g. olive oil, canola oil).

□ Animal oils (lard).

□ I don't use

24. What condiments do you use ?

□ Salt

□ Pepper

□ Vegeta, magic

□ Sweet paprika

□ Hot paprika

□ Garlic

□ Marjoram

□ Thyme

□ Oregano

□ Basil
